# Potential Facilitators of and Barriers to Implementing the MINI Robot in Community-Based Meeting Centers for People With Dementia and Their Carers in the Netherlands and Spain: Explorative Qualitative Study

**DOI:** 10.2196/44125

**Published:** 2023-08-02

**Authors:** Aysan Mahmoudi Asl, Suzanne Kouters, Álvaro Castro-González, Henriëtte Van der Roest, Manuel Franco Martin, Rose-Marie Dröes

**Affiliations:** 1 Psycho-Sciences Research Group of the Biomedical Research Institute of Salamanca Salamanca University Salamanca Spain; 2 Department of Clinical Psychology Faculty of Behaviour and Movement Sciences Vrije Universiteit Amsterdam Netherlands; 3 Robotics Lab Department of System Engineering and Automation Universidad Carlos III de Madrid Madrid Spain; 4 Department on Aging Netherlands Institute of Mental Health and Addiction (Trimbos Institute) Utrecht Netherlands; 5 Psychiatry and Mental Health Department Zamora Hospital Zamora Spain; 6 Department of Psychiatry Amsterdam University Medical Center Vrije Universiteit Amsterdam Netherlands

**Keywords:** dementia, meeting centers, mild cognitive impairment, social robots

## Abstract

**Background:**

Social robots, as a form of digital health technologies, are used to support emotional, cognitive, and physical care and have shown promising outcomes in enhancing social well-being in people with dementia (PwD) by boosting emotions, social interactions, and activity participation.

**Objective:**

The goal is to investigate the attitude of stakeholders and potential facilitators and the barriers to implementing the social robot MINI in community-based meeting centers (MCs) for PwD and carers in the Netherlands and Spain.

**Methods:**

Based on the British Medical Research Council guidance for process evaluation of the implementation of complex interventions and the model for tracing the facilitators of and barriers to the adaptive implementation of innovations in dementia care, an explorative qualitative study was conducted. Following the introduction of the MINI robot, 11 stakeholders were interviewed in 3 MCs in the Netherlands and 1 in Spain, as well as stakeholders in health and welfare organizations in both countries. In addition, 12 adults with dementia participated in focus groups. The data were thematically analyzed and narratively described.

**Results:**

Overall, the stakeholder opinion and interest in the MINI robot were positive. The most important (expected) facilitating factors mentioned by stakeholders appeared to be human resources, funding, the impact of the MINI robot on the users and programs of the MCs, characteristics of the innovation, and collaboration with other care and welfare organizations. However, the (expected) barriers mentioned concerned the physical context and functionalities of the MINI robot, the user context, and MC activity policies.

**Conclusions:**

The findings will inform professional stakeholders, such as MC directors and managers, as well as care and welfare organizations, on the practicality of using the MINI robot in MCs. Furthermore, our research will aid MINI robot developers in tailoring its features to PwD’s preferences and demands and MC policies, which will contribute to the MINI robot’s effective adoption and deployment.

## Introduction

Dementia has been identified as 1 of Europe’s most complex public health diseases, which impacted 14.1 million people in 2019, and has been highlighted as a public health priority [1]. As a response to providing social and health support programs for people with dementia (PwD) and reducing the adverse effects of dementia, the Meeting Centre Support Programme (MCSP) for PwD and their carers was set up in the Netherlands in 1993. Recently, the MCSP was adaptively implemented in several other European countries as well (the United Kingdom, Poland, Italy, and Spain) [2]. Currently, there are more than 180 MCs across the Netherlands and around 70 in other European countries. The MCSP has been proven to be effective in the case of psychosocial symptoms, such as mood and depression, inactive and unsocial behavior, and quality of life, as well as the carer burden and delayed nursing home admission of PwD [3-8].

Intelligent assistive technologies (IAT) are a growing body in dementia research [9]. “IAT” is an umbrella term including a range of digital devices, such as smart home systems, tablets, smartphones, wearable devices, and humanoid robots. According to a systematic review, IAT have been suggested to empower people in the mild-to-moderate stages of dementia, supporting their independence for a longer time [10]. Humanoid robots, as the third generation of social robots, communicate with humans through verbal and nonverbal interactions. They have the potential to improve the mood, social interaction, and activity participation of PwD [11]. However, due to the early stage of this field, few of them are fully developed and commercially available.

MINI is a humanoid robot recently developed at the University of Carlos III of Madrid. This social robot is designed to assist and accompany older adults in their daily lives at home or in a nursing facility and stimulate their cognitive functions. The robot can provide psychosocial and cognitive stimulation through games and cognitive tasks, in addition to services in the areas of safety, entertainment, and personal assistance.

Because the MINI robot may be valuable for the target group of MCs, we explored the interest of key stakeholders in MCs in the Netherlands and Spain, as well as possible facilitators of and barriers to the robot’s deployment in MCs.

Several frameworks and guidelines have been suggested in the literature to aid in the successful implementation of complex interventions. The Medical Research Council (MRC) published a framework for the development and evaluation of complex interventions and to help researchers with methodological difficulties [12]. The MRC also developed a guidance for process evaluation of the implementation of complex interventions, which focuses on the influence that contextual factors, implementation factors, and mechanisms of impact may have on the outcomes of interventions [13]. The effectiveness of an intervention can be limited due to flaws in proper implementation [14]. Hence, the MRC framework emphasizes the importance of intervention implementation ingredients, such as fidelity, dose, and reach, and addresses issues such as management, structure, support, and training to shape proper intervention conditions. The term “mechanism of impact” refers to how the proposed intervention brings about desired changes and how these outcomes might be repeated in future interventions of a similar nature.

Understanding the context in which a complex intervention takes place becomes important when external contextual elements may facilitate or impede proper implementation and as such may influence the intervention outcome.

Another theoretical model to evaluate the implementation of complex interventions was proposed by Meiland et al [15]. Their model is designed to trace the facilitators of and barriers to the adaptive implementation of innovative practices in dementia care, attuned to local and regional conditions [15]. Preconditions, as well as the phases of preparation, execution, and continuation of implementation, are covered by this theoretical framework. Preconditions refer to existing conditions in the context where the intervention will be implemented, including characteristics of the innovation, operational conditions, human and financial resources, and organizational conditions that can facilitate or impede the implementation of the intervention. In the preparation phase, the need for adaptively implementing the intervention is assessed and an inventory of the practical preparations required for successfully implementing the intervention is created and evaluated. Regarding the execution phase in which the intervention is started in practice, elements that interact with the high-quality execution of the intervention are evaluated. In the final phase, continuation, elements that contribute to an effective intervention are evaluated. The above-mentioned phases are further specified in the model on 3 levels: micro (user/primary process, eg, personnel, training, management, care provider, person with dementia, and informal carer), meso (collaboration between care providers/organizations, finances, division of tasks, collaboration with other organizations), and macro (structure of the care system, laws, regulations, and national and regional policies).

In this study, we used both the MRC guidance for process evaluation and the model to trace the facilitators of and barriers to adaptive implementation of innovative practices in dementia care in order to explore the facilitators of and barriers to the implementation of MINI in MCs for PwD and their carers. In this research, we did not include the continuation phase, since the robot has not yet been implemented in the MCs.

The research questions were:

- What are the potential facilitators of and barriers to the implementation of the MINI robot in MCs for PwD and carers in the Netherlands and Spain?
- Do any of the robot’s features need to be improved for successful implementation in MCs?
- What is the attitude of PwD and other stakeholders in MCs toward the MINI robot?

## Methods

### Study Design, Participants, and Setting

An explorative qualitative design, using one-off semistructured interviews with stakeholders other than PwD and focus groups with PwD, was used to identify potential facilitators of and barriers to implementing the MINI robot for PwD and people with mild cognitive impairment attending MCs in the Netherlands (N=3) and Spain (N=1). Participants were selected and recruited through the purposive sampling method. The participants interviewed in the Netherlands came from 3 MCs in the middle and west of the country: 12 people with mild-to-moderate dementia who were invited by the coordinator of the MCs to participate in the focus groups and 9 professional stakeholders, including the managers and activity therapists of the 3 MCs, the chair of the Amsterdam section of the Dutch Alzheimer Association, and a senior policy advisor and coordinator of the social support domain of the municipality of Amsterdam. In Spain, the manager of the MC and head of the Community Support Complex in Zamora participated.

### MINI Robot Presentation

**Figure 1:**
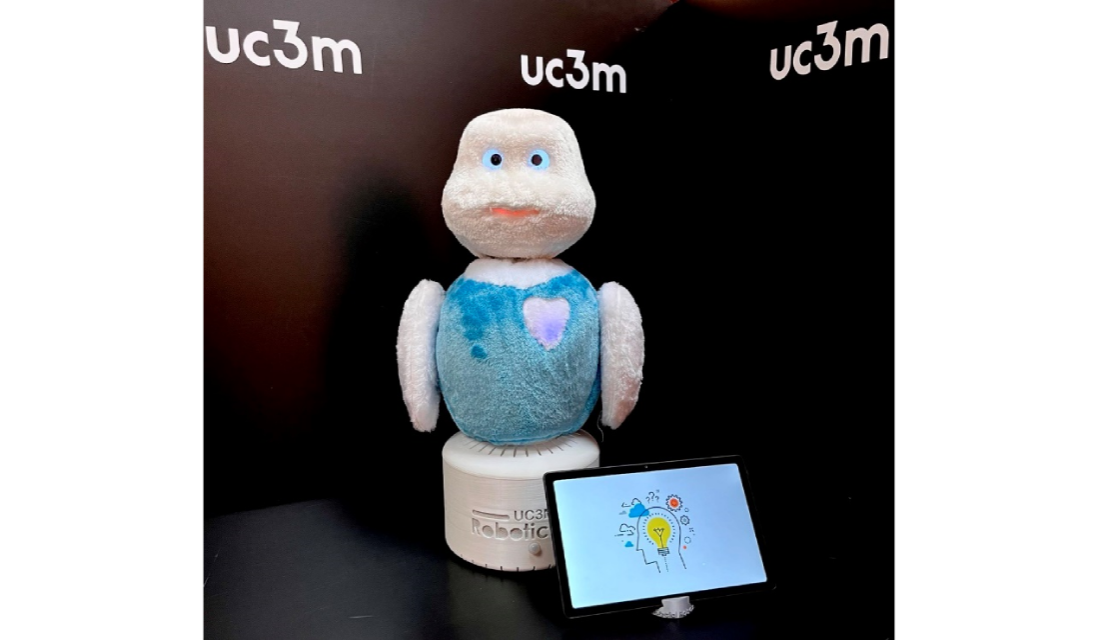
MINI robot developed by the University of Carlos III of Madrid (UC3M).

MINI autonomously communicates with humans through verbal and nonverbal interactions. It is a lightweight, small-size, stationary robot covered in plush fabric with an animal appearance. It is equipped with a microphone for speech-based communication, touch sensors in the shoulders and belly that allow the user to interact physically with MINI, an RGB-D camera to extract visual and depth information from the environment, organic light-emitting diode (OLED) eyes to express the robot’s emotional state, and a tablet that can work both as an input device (through fixed menus) and as an output device, displaying the content of the apps. A series of games and cognitive exercises are installed on the robot that can be extended and modified. MINI is set up to pose certain queries, obtain answers, and provide users with feedback on their performance [16-18]. Currently, MINI is in the prototype phase and programmed for the Spanish language only (see Figure 1).

An introduction video clip of the MINI robot was produced in collaboration with the robotic laboratory of the University of Carlos III of Madrid and translated into Dutch, adding a Dutch voice-over and subtitles, by researchers of the Amsterdam University Medical Center. The video provides information about the use and functionality of the MINI robot, as well as its software and application, and demonstrates a user interacting with the robot. This video was sent to participants via email as a YouTube video link before the interview day, accompanied by the study aim, a brief description of the MINI robot, and interview questions. The interviews were conducted in person, and participants were free to watch the video again before answering the questions.

**Figure 2:**
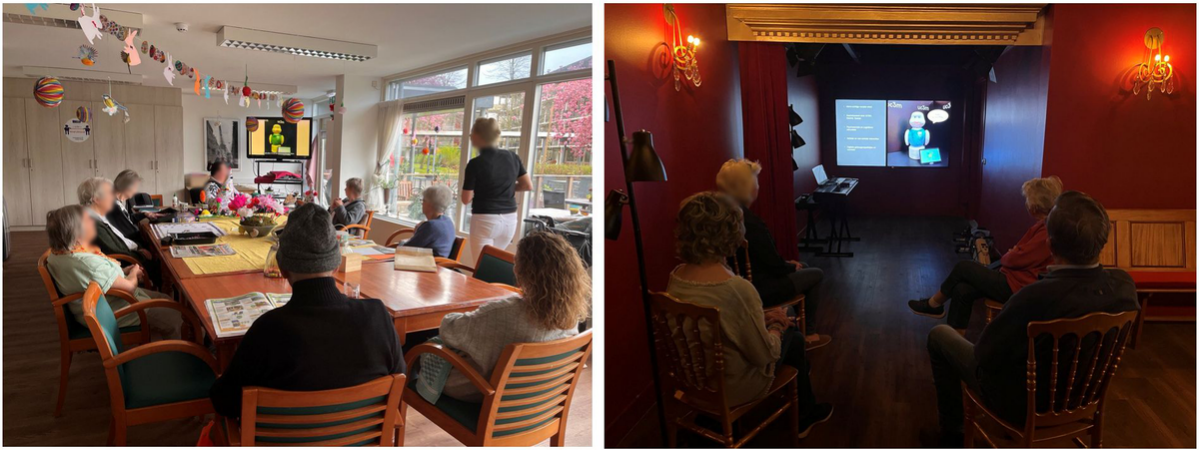
Presentation of the MINI robot to the PwD in the MCs. MC: meeting center; PwD: people with dementia.

For the PwD, the video was shown on a big-screen TV/projector screen during a focus group session, and general questions were asked about their opinions and attitudes toward the MINI robot (see Figure 2).

### Data Collection

**Figure 3:**
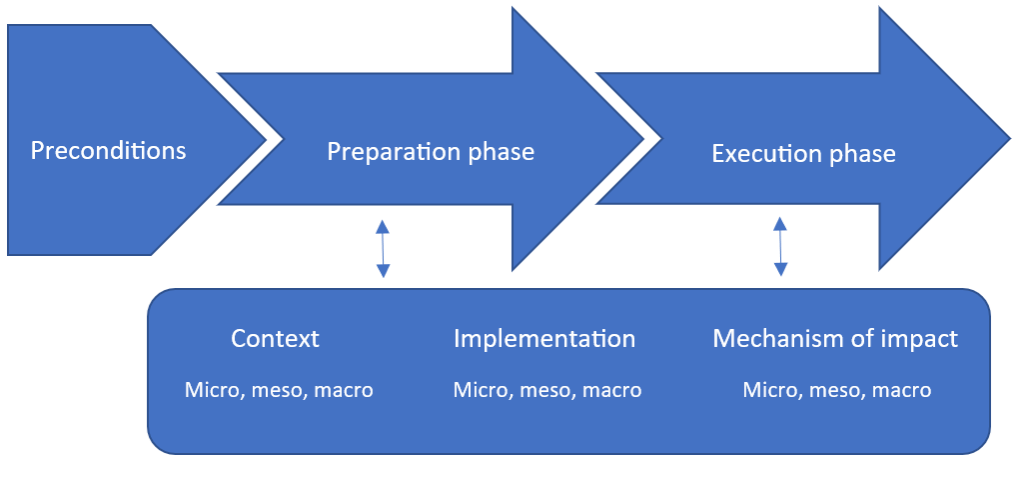
Combined model for tracing the facilitators of and barriers to implementation of the MINI robot based on the Medical Research Council’s guidance for process evaluation.

The interview questions were created based on the MRC guidance for process evaluation and Meiland et al’s [15] theoretical framework to trace facilitators and barriers regarding preconditions and the preparation and execution phases of implementation at micro-, meso-, and macrolevels, including contextual factors, implementation factors, and mechanisms of impact. Figure 3 demonstrates how the 2 models were combined to create a theoretical framework of our explorative study. The semistructured interview questions for the stakeholders other than the PwD are presented in Multimedia Appendix 1.

Two interviewers, the main researcher of the study (author AM) and a local researcher in the Netherlands (author SK), interviewed the stakeholders individually and took notes (quotes) of what was stated, and all interviews were audio-recorded.

In the focus groups, the PwD discussed their general attitudes toward the implementation and application of the MINI robot in the MCs. The local researcher (SK) interviewed and moderated the focus group sessions, and the sessions were audio-recorded.

### Data Analysis

The interviews were anonymously recorded using the iPhone 13 voice recording app. The local researcher (SK) transcribed the audio recordings and translated them into English. The main researcher (AM) followed the same procedure for the interviews in Spain. Codes for facilitators and barriers regarding preconditions, preparation and execution phases, level (micro, meso, macro), and factors associated with the context, implementation, and mechanism of impact were created. Subsequently, the study supervisor (author RMD) and main researcher (AM) independently coded 3 interview transcripts, reviewed disparities, and discussed them until consensus was reached. The other transcriptions and meeting notes were then further coded thematically by the main researcher (AM) using QSR NVivo 12 software, and potential facilitators and barriers were summarized. The complete list of codes is presented in Multimedia Appendix 2.

### Ethical Considerations

Ethical approval was not sought for this study. This aligns with the Medical Research Involving Human Subjects Act (Wet Medisch wetenschappelijk onderzoek met mensen, WMO) in the Netherlands [19] as interview studies are beyond its scope. Additionally, no personal data were collected. All participants were fully informed of the study and what was being asked of them. Verbal informed consent was obtained from individuals before the study.

## Results

### Participants

In total, 11 interviews were conducted with 11 stakeholders: 4 (36%) MC managers, 4 (36%) activity therapists of the MCs, the chair of the Amsterdam section of the Dutch Alzheimer Association (n=1, 9%), a senior policy advisor/coordinator of the social support domain of the municipality of Amsterdam (n=1, 9%), and the head of the psychiatry unit of the Community Support Complex and manager of the MC in Zamora (n=1, 9%). Additionally, 12 Dutch PwD attended the focus group discussions.

### Facilitators of and Barriers to Implementation of the MINI Robot

In this section, a summary of the facilitators and barriers in existing preconditions as well as those associated with the 3 process evaluation components (ie, contextual factors, implementation factors, and mechanisms of impact) in both preparation and execution phases are outlined (see Tables 1-4).

**Table 1:** Existing conditions facilitating or impeding the implementation of the MINI robot.

Precondition	Facilitators	Barriers
Characteristics of the innovation	Entertaining (M1^a^) Improving self-esteem (LG^b^) Fun and enjoyable (M1) Reminiscence activity (M1) Stimulating and activating (A^c^) Reduces the care burden of caregivers and professionals (M2^d^) Complementary/additional activity (M1, A)	No substitution for the human being Individualized use May substitute human interactione Constant need for supervision^e^
Operational preconditions	N/A^f^	Need for a technical person (M1)
Human and financial resources	Existing budget from the municipality (M1) Decision-making inside the organization (M1) No need for approval from higher-level parties in the organization (M1) Available donations from tech companies (M1) Available staff and volunteers (M1)	Busy activity therapist (A)
Organizational conditions	No need for insurance for using the robot in the MC (M1)	No quiz-like activities and games policy of the MC (M1, A) Permission required from the directors (M1)

^a^M1: meeting center (MC) manager.

^b^LG: local government.

^c^A: activity therapist.

^d^M2: manager/director of other care/welfare organization.

^e^As stated by people with disabilities (PwD).

^f^N/A: not applicable.

**Table 2:** Contextual factors: potential facilitators and barriers, and recommendations for improvement.

Factor	Facilitators	Barriers	Recommendations for improvement
Hardware and physical context of the MINI robot	Cute appearance (M1^a^)	It should look like a pet or human but not in between (A^b^) Monotonous voice (M1) 2 user interfaces making it complex to use: the robot and the tablet (M1) Not a cute face (M1) Not a nice appearance^c^ Childish appearance^c^	Moving eyes (M1) Expressing emotions (eg, laughing at jokes; M1) Less toy-looking (A) Change the appearance (M1) Should look like a child or young adult but not too childish or baby-looking (M1) Huggable and soft touch^c^ (A) 2 versions of the robot’s look: puppet and adult look (A) Use of special fabric that cleans itself (A) Changeable clothing (A) Clear voice^c^ Slow and short sentences^c^ (M1) Use of bright colors^c^ (M1) Add some hair^c^
Functionalities and software context of the MINI robot	Possibility to add new apps (M2^d^) Interesting software (M1, M2) Not too difficult (M1)	Too many options on the tablet screen could be confusing (M1, A) Features of the robot for individual settings (M1) No reaction to people’s emotions (A) Too much explanation by talking (M1) No feelings^c^	Expressing emotions (eg, lighting to the jokes) (M1) Medication reminder (LG^e^, M2) Make the games more complicated (M2) An alarm of accidents, fire, etc (M2) Simple robot without a need for too many explanations (M1) Robot in a group setting acting as a member of the group (M1) Pictures from the local city (A) Support and guidance in performing daily tasks (LG)
Users of MCs	N/A^f^	Higher functions of the brain damaged so not suitable for people in MCs (M1, A) Repetitions of instructions needed by users (A)	N/A
Setting and activities of MCs	N/A	No quiz-like activities and games policy of the MCs (M1, A) Not playing games in the MCs (main activity is to socialize) Normal activities such as music and art (M1, A)	N/A
Other settings	Could be helpful in other settings such as nursing homes or private homes where people are truly alone Can get used to the robot in MCs and then want to have it at home (M2)	N/A	N/A

^a^M1: meeting center (MC) manager.

^b^A: activity therapist.

^c^As stated by people with disabilities (PwD).

^d^M2: manager/director of other care/welfare organization.

^e^LG: local government.

^f^N/A: not applicable.

**Table 3:** Implementation factors: potential facilitators and barriers.

Factor	Facilitators	Barriers
Insurance	Insurance probably possible for some people (M1^a^)	Insurance is difficult to reach (M1)
Human resources and supervision	Volunteers of Digi Café can help with implementation (M1) Clear instructions for using the robot (M1) Getting used to the robot (M1)	Need for support from a caregiver besides the robot (A^b^) Need for a technical person to fix the robot (M1)
Funding	Many organizations gifting to elderly adults (M1) A medical general practitioner (GP) can recommend a technology (M1) The university can promote new interventions (M1) The municipality can provide funding (LG^c^) The care organization can provide financial resources (M1) The social support domain of the municipality of Amsterdam supports financial aid for such projects (LG)	The technology must be necessary to get insured (M1)
Effectiveness	Evidence of efficacy necessary (M1) The MINI robot in line with the Amsterdam Vital and Healthy program (LG)	N/A^d^
Collaboration of organizations	Collaboration with welfare and health organizations can help implement the MINI robot (LG) Doe-Mee Huis^e^ and Bakkershuise can help with implementation (M1) Collaboration with the Alzheimer Association can help implement (M2^f^)	N/A

^a^M1: meeting center (MC) manager.

^b^A: activity therapist.

^c^LG: local government.

^d^N/A: not applicable.

^e^Community-based day centers.

^f^M2: manager/director of other care/welfare organization.

**Table 4:** Mechanism of impact: facilitators and barriers.

Factor	Facilitators	Barriers
MCs^a^	An MC with a robot would be interesting (M1^b^) The robot can be attractive and change the routines of the MC (M1) Fun and promoting social interactions (M1) Engaging the person in the group could affect their mood (M1) They will have more conversations (about the robot) with their partner in the MC (M1) Helpful to relax and make jokes (M1) Fun and entertaining (M1) Helps against cognitive deterioration (M1) Helps to stay active (M1)	Will not decrease the burden on professionals/caregivers (M1, M2^c^) Will not decrease loneliness (M1, M2)
Other settings	Social interactions (M1) People will have more conversations (about the robot activities) with their partners at home (M1) Could be helpful for people living alone (M1, M2)	N/A^d^

^a^MC: meeting center.

^b^M1: MC manager.

^c^M2: manager/director of other care/welfare organization.

^d^N/A: not applicable.

#### Preconditions

Although the stakeholders’ views on the features of the MINI robot were somewhat divergent, most of them supported the use of the MINI robot in MCs or other environments, such as private or resident homes. Several existing facilitators were noted in terms of human and financial resources. A few impeding factors were noted for operational and organizational conditions. Table 1 presents the existing facilitators and barriers for the 4 precondition components.

### The Microlevel

#### Introduction of the MINI Robot (Preparation Phase)

Obtaining consent from the directors of the MCs’ to implement the MINI robot for PwD visiting the MCs may be considered the first step in the preparation phase. However, the majority of MC managers claimed that they could make this decision on their own without seeking permission from their superiors. The stakeholders suggested various ways to inform PwD about the new MINI robot intervention, including social media groups, educational events, phone calls, and emails. The chair of the Alzheimer Association suggested the MINI robot be presented at conferences first so that various stakeholders may become familiar with it.

It’s very important to see how you can get people on board so that they don’t immediately put the brakes on.

Policy advisor of the social support domain of the municipality

#### Human Resources and Training (Preparation Phase)

Every member of the MCs’ staff may participate in the MINI robot’s implementation, depending on their willingness to use such technologies in the MCs. The MC managers stressed the importance of training their staff as facilitators of the intervention so that they may manage the robot and provide users with instructions on how to use it. They all believed that users would not be able to communicate with the robot on their own.

I cannot leave those people alone. Because they don’t know what to do. You always have to be with them.

MC activity therapist

#### Frequency of Use (Execution Phase)

The management and activity therapists largely agreed to use the robot 3 days per week or as often as the users desire to interact with it. One of the MC managers said that it might take several weeks to get users accustomed to the robot before using it regularly.

#### Adjustments to the Robot (Preparation Phase)

Regarding the robot’s software, hardware, and functions, people had a wide range of comments to improve its features (see also Table 2). All interviewees recognized the opportunity for development in the aforementioned areas.

Regarding the software interface, it was frequently noted that the ease of use and simplicity of the functions would make it easier for PwD to interact with the robot, because they are not accustomed to using such technologies and could become confused if given too many options, lengthy usage instructions, or a cluttered screen interface. The robot would integrate effectively into the MCs if it could speak and converse socially, respond to people’s emotions, and act naturally.

It would be great if the robot reacts to the people when, for instance, they say, I’m feeling sad today. I’m feeling really sad, that he reacts to that.

MC manager

It would be helpful if the robot could express emotions, for instance, that he can laugh.

MC manager

I think it would be nice when it can have social talks about anything.

MC manager

Since the MCs’ support program is group oriented, individual activities do not often take place. One manager saw the current features of the robot interacting with 1 user in an individual setting as a barrier to implementation and suggested adapting the robot for a group setting.

The robot will be an individual activity unless you could use it in a group setting for at least 2 people, not just 1 individual!

MC manager

Concerning the robot’s hardware and appearance, the interviewees found it attractive but believed there is a need for improvement to make it more acceptable to older adults (see also Table 1). A few interviewees were not sure whether the robot is an agent that resembles a human or an animal and thought the appearance was somewhere in the middle. Additionally, some people preferred the robot to look like a young adult and disliked the childish look. Moreover, one of the activity therapists suggested that the robot’s voice be clearer and slower, communicating through brief sentences. Another activity therapist recommended using special fabric for the robot’s clothes that cleans itself and does not require regular washing, or even changeable clothing, ensuring safe hygiene if people touch and embrace the robot (see Table 1).

#### Impact of the Robot on the MCs (Execution Phase)

Except for 1 MC, all other MCs recognized the MINI robot’s potential to impact them. Depending on how the robot is programmed, it can stimulate cognitive functions, keep people more alert and active, and even improve their mood. The interactions with the robot were viewed by some of the stakeholders as a fun, entertaining, and engaging activity that might even encourage dialogues between participants, as well as dialogues between participants and caregivers/therapists. One of the MC managers was hesitant to deploy the MINI robot because it did not seem to fit with the MC’s norms and regular activities: they do not undertake activities that focus on stimulating high-level brain processes, as they do not want PwD to be confronted with their disabilities or fail. A few of the stakeholders thought that the robot could play a role in alleviating loneliness in persons who live alone in their private homes.

Getting less lonely in the MC is not expected, but I do think it may result in less deterioration…I do think that helps…It’s especially important to keep people stay active.

MC manager

A few of the stakeholders did not expect that the MINI robot would benefit care and welfare professionals; however, there were many supportive remarks from the rest of the stakeholders: The MINI robot intervention might provide an extra variety of activities in the MCs and may reduce the workload and time spent by the professionals and caregivers with PwD. It could encourage social interaction and conversations between PwD and formal/informal caregivers in the MCs, according to 1 of the managers.

Robots cannot replace the therapies but can be a complement for doing all their activities in another way.

MC manager

### The Mesolevel

#### Collaborations With Other MCs and Health Care/Welfare Organizations (Preparation and Execution Phases)

The implementation of such technologies could be promoted by collaborations outside the MCs through the university and even a general practitioner. In terms of providing human resources and encouraging implementation, Doe-Mee Huis, Bakkershuis, and Digi Café (community-based day centers) could serve as facilitators. In Spain, a collaboration between the MC, the Alzheimer Association, and other foundations may help with the robot’s implementation.

### The Macrolevel

#### Financial Resources (Preparation and Execution Phases)

Several external resources were discussed in the interviews to obtain sufficient financial resources in order to purchase and implement the MINI robot. However, some of the stakeholders highlighted that financial support would be contingent on the robot’s proven effectiveness and necessity. The municipality, the social support domain of the municipality, and the care organization exploiting the MCs were mentioned in the Netherlands as potential sources of financial support. In addition, as a facilitator for introducing such technologies, 1 of the stakeholders pointed to the Amsterdam dementia-friendly city program and that nowadays people are more open to using such technologies for PwD. In addition, a manager confirmed that some corporations provide such technologies as a gift to PwD. One of the managers also suggested the option of splitting the cost of the robot across several MCs or departments of the care organization by sharing it.

That’s also a bit the trend in the Netherlands now—that you connect care and welfare—to bring down the expensive care costs…then people can live with some quality of life at home, for as long as possible.

Social support policy advisor

In Spain, supported by social security, the majority of health care expenses are covered by the government, and in the event of proven effectiveness, funding can be received from the government and various foundations.

#### Coverage of Health Insurance (Preparation Phase)

Although there is no need for the users of MCs to pay separately for using a robot there, in the case of demonstrated effectiveness, as noted before, health insurance may be willing to cover the costs of using the robot in both MCs and the private homes of PwD.

It is always interesting to see in pilots how things work…our city is also very open to that—to try new things out.

Social support policy advisor

#### Information Available on Health Policy or Legislation (Preparation Phase)

None of the stakeholders were aware of any existing policy in the Netherlands or Spain that supports the application of technologies such as social robots for PwD.

#### Focus Group Interviews With PwD

PwD in the Dutch MCs had various reactions to the MINI robot, although most of them had doubts about using it. They were unaware of all of the robot’s specific capabilities and applications, since only a few functions of the robot were demonstrated in the presentation video. Some thought the robot was a nice and adorable doll machine to have in the MC. Some were skeptical of deploying it, seeing it as something for the younger generation or those in the early stages of dementia. They said they had never used such technology before, and several even claimed they had no idea how to operate smartphones. One participant was concerned that human engagement might be replaced by robot interaction:

I am afraid that you will lose contact with real people a bit if you constantly fixate on the robot…it helps you, it looks nice, and then what? Will you then get on better with other people?

One of the participants emphasized that they would rather play a normal game in the MC instead of playing with a robot.

Playing games! People can do that anyway; they don’t need the robot for that.

Regarding the robot’s looks, there were differing opinions: some people thought the robot was attractive, while others did not. They also liked the robot to speak in short, concise sentences and to speak slowly.

## Discussion

### Principal Findings

This study provides insight into facilitating and impeding factors for the implementation of the social robot MINI in MCs for PwD and their carers in the Netherlands and Spain. Several influencing factors were found in preconditions, and the preparation and execution phases at 3 levels (micro, meso, and macro), associated with contextual factors, implementation factors, and mechanisms of impact. The most important (potential) facilitating factors noted by the stakeholders seemed to be human resources, funding, the impact of the MINI robot on the users and program of the MCs, characteristics of robotic interventions, and collaboration with other organizations. The (potential) barriers mentioned concerned the physical context and the functionalities of the MINI robot, the user context, and activity policies of MCs.

### Facilitators

The overall characteristics of the robotic intervention were well recognized by the stakeholders as potentially benefitting PwD in several ways: by keeping them active and alert, by entertaining them and stimulating their cognitive functions, and, most importantly, by having fun and participating in enjoyable activities. Based on a recent systematic review, social robots might benefit people with mild-to-moderate dementia with psychosocial symptoms and agitation [20]. Another scoping review revealed the impact of social robots on the mood and emotions, activity participation, and social interaction of PwD [11]. The stakeholders also saw the potential for loneliness relief by using the MINI robot among PwD living alone. However, research in this area is rare, and most existing pilots have been conducted in laboratory settings.

The positive views on the potential impact of the MINI robot intervention in the MCs could be supported by recent studies on other social robots in similar environments in daycare centers for PwD. A recent randomized controlled trial [21] on the PARO robot with PwD in daycare centers showed improved facial expression and better communication with staff. This was 1 of the most commonly highlighted impacts in MCs by professional stakeholders that may promote interaction between MC users and staff and a pleasant and pleasurable variety of activities.

Another facilitating aspect that was frequently mentioned regarding the preconditions and the preparation phase was human resources. Participants felt that almost all the MC staff, including activity therapists, volunteers, and internship students, could facilitate the MINI robot’s implementation and promote its use in the MCs. To modify and tailor the robotic intervention, the background and training of the intervention facilitators should be considered.

Considering the high cost of purchasing social robots and maintenance [22], the available and possible funding resources to support the implementation of the MINI robot in MCs were conceived as a key facilitator. The stakeholders believed that there is a budget available to purchase digital technologies, as well as organizations/institutions to refund or donate the MINI robot. In the literature, the high cost of social robots has been widely acknowledged as a practical issue by users, family members, stakeholders, organizations, and researchers [23-26]. Thus, by sharing a social robot in daycare centers, equal access for older adults and PwD can be promoted [27].

### Barriers

Regarding the characteristics of the MINI robot, hardware and software were much discussed. Most professional stakeholders and PwD considered the MINI robot’s hardware and software to be a hindrance. In terms of the appearance and physical body of the robot, there is room for modifications/improvements to make it more attractive and better usable. One of the studies on social robots [28] showed that the users appreciated both the mechanical human-like and the mechanical animal-like appearance of the robots. This might support the opinion of some of the stakeholders who did not like the current ambiguous appearance of the MINI robot—neither being animal nor human but rather a mixture. Some stakeholders voiced concerns about the monotonous and rather fast-speaking robot and perceived it as a machine-like agent that may raise both usability and acceptability challenges for PwD who are trying to follow the dialogue. In a recent study on the Pepper robot [29], considering user opinions of the tablet interface, the researcher recommended large buttons and text sizes for easy usability for older adults. To ensure trouble-free interaction, they also suggested a simple and clear tablet user interface.

The main impediment in 1 of the MCs was the position on games and activities for PwD. The MC staff was opposed to using any form of quiz-like game or other activity that requires semantic memory in order to prevent PwD from having to confront their disease and failure. However, the rest of the professional stakeholders were optimistic about the robotic intervention to keep PwD active and alert. In a recent systematic review, the negative attitude of professionals and relatives was shown as a barrier to social robot implementation [30].

### Limitations and Strengths of the Study

This study has several limitations that must be noted. First, the MINI robot’s capabilities were introduced and demonstrated through a video clip, which may have limited the understanding of the robot’s characteristics and functioning. A video presentation may not be able to replace hands-on encounters with the robot and may impact attitude and trust in the robot. Second, the video clip did not show the entire sequence of games and apps that the robot provides. This may also have had an impact on the stakeholders’ perceptions. Third, only an inventory of *potential* facilitators and barriers could be made, as the MINI robot was not yet implemented in the MCs. This requires cautiousness regarding the study’s findings. Study of the facilitators of and barriers to implementing the MINI robot should therefore be repeated after improving the prototype and the actual physical implementation. Fourth, there were no participants with dementia in Spain and the sample size for PwD in the Netherlands was small. In Spain, currently, only 1 MC has been established. People in the MC in Spain at the time of the study were involved in another research experiment, making a focus group interview with them practically impossible.

The strengths of the study were that we interviewed a wide range of stakeholders, ensuring that all points of view were well represented. The interview scheme was built on 2 theoretical frameworks, which ensured that all components necessary for successful implementation were addressed in the interviews.

### Scientific, Clinical, and Societal Relevance of the Study

The scholarly significance of this study stems from the fact that it is the first to investigate the potential facilitators of and barriers to adopting the MINI robot in MCs for PwD and carers in the Netherlands and Spain. This benefits robotic intervention research and clinical practice in 2 ways: First, to effectively install the robot and enhance its usability and acceptance as viewed by end users, the robot’s creators need feedback from both professional stakeholders and PwD about the MINI robot itself and its functionalities. Second, by identifying possible facilitators of and barriers to the adoption and implementation of the MINI robot in an early phase, the directors and managers of MCs may facilitate the process later. Our study discovered that most professional stakeholders and PwD perceive the robotic intervention favorably and feel it is feasible to apply the MINI robot in MCs in the Netherlands and Spain. However, we also uncovered a few roadblocks, most of which involve the MINI robot’s characteristics, that can be addressed. The societal relevance of this study is that it provides a better understanding of the implementation of the MINI robot or similar social robots in the local context in MCs in the Netherlands and Spain. Based on current research on the influence of social robots in daycare centers on promoting activity participation, emotions, engagement, and social interaction, the successful implementation of the MINI robot will contribute to the enhancement of PwD’s mental and social health.

### Recommendations for Future Studies

Continued study on the MINI robot’s deployment and influence on PwD in MCs is crucial to establish its usability, feasibility, and efficacy in a real-world context. To achieve this goal and maximize the benefits of robotic interventions for PwD, the MINI robot engineers and researchers need to tailor the robotic platform to the end users’ needs and preferences, the dynamics of group interactions, and the MCs’ activity policies and settings.

### Conclusion

This study provides insight into the facilitating and impeding factors for the MINI robot’s implementation in MCs in the Netherlands and Spain. The findings will inform professional stakeholders, including the directors and managers of the MCs and care and welfare organizations, about the feasibility of the MINI robot in MCs. Moreover, our research will assist the MINI robot developers in adapting its characteristics to the preferences and demands of PwD as well as to the MCs’ policies, which will contribute to the effective adoption and implementation of the MINI robot.

## Appendix

Multimedia Appendix 1 Interview questions.

Multimedia Appendix 2 List of codes.

## Abbreviations

IAT: intelligent assistive technologies
MC: meeting center
MCSP: Meeting Centre Support Programme
MRC: Medical Research Council
PwD: people with dementia
