# Supplementary material for: Potential Facilitators of and Barriers to Implementing the MINI Robot in Community-Based Meeting Centers for People With Dementia and Their Carers in the Netherlands and Spain: Explorative Qualitative Study
Source: J Med Internet Res. 2023 Aug 2;25:e44125. doi: 10.2196/44125 (PMC10433023; doi:10.2196/44125)
Supplement: Multimedia Appendix 1 [file jmir_v25i1e44125_app1.docx]

| Interview Questions | | |
| --- | --- | --- |
| Conditions | | Preparation phase |
| **Area** | **Ask** | **Micro**   1. Where would you get the funding for this intervention? (M1) 2. What do you need to prepare for the robot implementation? (M1, C) 3. What adjustments do you think you need to make to the intervention so that it will work effectively in your meeting center? (M1, C, A, T) Remember:   O Change software content/activities.  O Adjusting the hardware.  O Difficulty of the games and exercises   1. Could someone (or a team) from outside your organization help you implement the intervention? (C, M1) 2. Who could be responsible for and involved in the implementation of the robot in daily practice? (M1, C, A) 3. How would you inform the participants and caregivers of your meeting center and caregivers about the implementation of this new intervention? (M1, C)   O Information meetings.  O Email.  O Phone calls  O Newsletter  **Meso**   1. Would a collaboration between the meeting centers and other healthcare organizations/systems help to implement the robot (jointly)? (M1, M2, LG)   Yes, namely …………………………  New    **Macro**   1. What needs to be done to obtain approval from your organization for the implementation of the robot? (C, M1, M2)   Is there anything else you would like to discuss or comments you would like to make about the robot or its implementation in meeting centers? |
| **Features of the intervention** | 1. What do you think of the intervention? (All stakeholders) 2. Would deploying the robot in your meeting center be of added value for the participants or the healthcare professionals? Yes, No, If so, in what sense?   A) For participants:  O participating in a fun activity  O memory training  O amusement  O social interaction  O having fun  O else …………………………  B) For healthcare providers:  O additional range of activities  O getting familiar with ICT  O else ………………………….  If not, why not?   1. Do you think this intervention fits the interests/needs/capabilities of the participants? (All stakeholders) 2. What do you think of the complexity of the intervention/robot for the participants (A)   O Simple O Easy to learn O Complicated O Very complicated   1. What do you think of the quality of the intervention/robot? (A, M1, M2T)   O Poor O Moderate O Good O Very good   1. Do you think the intervention in the meeting center will have an effect on the participants? (A, M1) (if no, see also III)   Yes No  Yes, namely:  O improved memory  O more active/alert  O less lonely  O more social interaction/  O improved mood   1. How well does the intervention match the values ​​and norms within the meeting centre? (A, M1)   O Not at all O Partly O Good O Very good.   1. Do you think the intervention will/can replace or supplement an existing program component, activity or process? (A, M1)   Oh yes, namely …………  Oh no |  |
| **Time and other organizational conditions** | 1. How much time do you think it will take for the full implementation of the robot in the meeting center, taking into account all organizational procedures to be followed? (M1, T)   O Less than 3 months O 3 to 6 months O More than 6 months   1. How often do you plan to use the robot in the meeting center?   O Less than 3 days a week O More than 3 times a week O Every day |  |
| **Financing and personnel** | 1. Which people in your organization do you think could play an important role in the implementation of the social robot? (M1, C)   O Manager organization  O Activity Therapist  O Program coordinator  O IT department   1. Do you expect to have or be able to acquire sufficient financial resources to implement and offer the intervention? (M1) (if No, I) 2. Do you think health insurance covers the use of the robot (as an aid)? (M1, M2, LG) |  |
| **Organizational conditions** | 1. What kind of health policy or legislation do you think applies to (co-)finance implementation of such technologies in the meeting center? (M1, M2, LG) |  |
| Remark. C: program coordinator, A: activity therapist, M1: meeting center manager, M2: manager/director other care/welfare organization T: ICT specialist, LG: local government | | |
