# Supplementary material for: Potential Facilitators of and Barriers to Implementing the MINI Robot in Community-Based Meeting Centers for People With Dementia and Their Carers in the Netherlands and Spain: Explorative Qualitative Study
Source: J Med Internet Res. 2023 Aug 2;25:e44125. doi: 10.2196/44125 (PMC10433023; doi:10.2196/44125)
Supplement: Multimedia Appendix 2 [file jmir_v25i1e44125_app2.docx]

**List of Codes**

**Precondition (A)**

- If the intervention fits the interests/needs/capabilities of the participants (precondition/characteristic of the intervention) A-1
- If the intervention matches the values ​​and norms within the meeting centre (precondition/characteristic of intervention) A-1
- The intervention will/can replace or supplement an existing program component or activity (precondition/characteristic of the Intervention) A-1
- Complexity of the intervention (precondition/characteristic of the Innovation) A-1
- Quality of the intervention/robot (precondition/characteristic of the Innovation) A-1
- Added value for the participants (precondition/characteristic of the innovation) A-1
- Added value for the healthcare professionals (precondition/characteristic of the innovation) A-1
- Time required for the full implementation (precondition/time and other operational preconditions) A-2

**Preparation phase (B)**

- Adjustments to the robot that make it more effective in the meeting center (preparation phase/micro/context) B-1.1
- The way of informing people about the robot (execution phase/ micro/ implementation) B-1.2
- Preparation for implementation (preparation phase/micro/meso/maco) B-1.2, B-2.2, B-3.2
- What needs to be done to obtain approval (preparation phase/meso/context) B-2.1
- Collaboration between the meeting centers and other healthcare organizations/systems (preparation and execution phase/ meso/context) B-2,1, C-2.1
- Ability to acquire sufficient financial resources (preparation and execution phase/meso/implementation) B-2.2
- People from outside that can help with implementation B-2.2, C-2.2
- Responsible people for the implementation (preparation and execution phase/meso/implementation) B-2.2, C-2.2
- Coverage of health insurance (preparation and execution phase/macro/Implementation) B-3.2
- Information on available health policy or legislation for implementing such technologies in meeting centers (Preparation and Execution phase/macro/context) B-3.1, C-3.1

**Execution phase (C)**

- Effect of the robot on the participants of the meeting center (execution phase/micro/mechanism of impact) C-1.3
- Frequency of implementing the robot (execution phase/micro/implementation) C-1.2
- People in your organization who could play an important role in the implementation (execution/micro/implementation) C-1.2
- People from outside that can help with implementation (preparation phase and execution phase/ meso/implementation) B-2.2, C-2.2
- Responsible people for the implementation (preparation and execution phase/meso/implementation) B-2.2, C-2.2
- Collaboration between the meeting centers and other healthcare organizations/systems (preparation and execution phase/ meso/context) B-2,1, C-2.1
